# Heterogeneity in Price Elasticity of Medicine Demand in China: Moderate Effect From Economic Incentive and Quality Difference

**DOI:** 10.3389/fphar.2021.688069

**Published:** 2021-08-02

**Authors:** Mingyue Zhao, Peng Nie, Jing Wu

**Affiliations:** ^1^ The Department of Pharmacy Administration and Clinical Pharmacy, School of Pharmacy, Xi’an Jiaotong University, Xi’an, China; ^2^ The Center for Drug Safety and Policy Research, Xi’an Jiaotong University, Xi’an, China; ^3^ School of Economics and Finance, Xi’an Jiaotong University, Xi’an, China; ^4^ School of Pharmaceutical Science and Technology, Tianjin University, Tianjin, China

**Keywords:** price elasticity, demand, price, quality, profit incentive

## Abstract

**Objectives:** Previous studies have shown a wide range of drug price elasticity, but the price response to demand among various therapeutic drug categories and drug types (generic/originator) is still unexplored in China. This study estimates the price elasticity of medicine demand with regard to quality differences, unfair competition, and a regulated market.

**Methods:** Product-level data on anti-tumor, cardiovascular disease (CVD), and antimicrobial drugs were collected from the Tianjin Urban Employees’ Basic Medical Insurance database (2008–2010). The moderating effects of quality, profit incentive, and illegal rebates are considered in a dynamic panel model.

**Findings:** Our results suggest that the price elasticity of drug demand varies across drug categories, with least elasticity for anti-tumor drugs and most elasticity for CVD drugs (−0.192 for anti-tumor drugs vs. −0.695 for antimicrobials vs. −1.100 for CVD drugs, *p* < 0.01). Moreover, the absolute value of price elasticity of generic drugs is higher than that of originator drugs in anti-tumor and CVD therapeutic classes (interact: 0.716 for anti-tumor; -0.630 for CVD, *p* < 0.001). We believe that quality difference plays a dominant role in the interaction between quality and illegal rebates for these two kinds of generic drugs. In the antimicrobial sub-group, the absolute value of price elasticity of generic medicine is lower than that of originator drugs. We believe that, owing to the high level of unfair competition among enterprises, the role of illegal kickbacks is dominant, which reduces the price elasticity of demand for generic antimicrobial drugs.

**Conclusion:** Our study provides an overview of the result of interaction between quality and illegal rebates in different medicine markets in China and shows that disease type is a primary factor that impacts price elasticity.

## Introduction

By 2023, it is estimated that global spending on medicines if left unchecked will reach US$1.5 trillion growing at an annual compounded growth rate of 3–6% (IQVIA, 2019). This is particularly true in China, where drug expenditure accounts for 40% of total health expenditure, far beyond the OECD average of 19% (National Health Commission of the People’s Republic of China, 2015). Prior to 2015, there was volume-based agreement for medicine covered by the UEBMI^1^ in China. However, a new pharmaceutical pricing reform was introduced in 2015. In the new reform, for off-patent drugs (originators) and generics, a new volume-based purchasing (VBP) policy was launched to reduce prices and promote generic substitution^2^ in China. The price–volume linkage has been established through the VBP policy. Thus, achieving accurate estimates of the price sensitivity of demand is crucial for controlling medicine expenditures and predicting medicine shortages under the current policy framework.

Various studies have shown that drug demand elasticities change in a wide range from −1.72 to −0.15 (Hughes and Mcguire, 1995; Gemmill et al., 2007; Chandra et al., 2010; Fiorio and Siciliani, 2010; Liu et al., 2012; Chandra et al., 2014; Yeung et al., 2018). Goldman et al. (2004) and Landsman et al. (2005) studied price responsiveness in different therapeutic drug categories. They conducted regression-type analyses using pharmacy claims data combined with cross-sectional studies on variations in health plan benefits designs. They found that drugs for chronic conditions are less price-sensitive (−0.1–−0.2) than drugs for acute conditions (−0.3–−0.6). Liu et al. (2012) also examined the drug market in Taiwan using product-level data. They showed that the demand for brand-name drugs is more sensitive to price changes than the demand for generic drugs (−1.721 vs. −0.549), although the opposite is true in the United States (Ellison et al., 1997; Frank and Salkever, 1997). Heterogeneity in price elasticities of demand has been attributed to different health insurance systems, data types, analytic methods, and diversity in the studied sub-populations. However, very few studies have examined the price elasticity of drug demands for different diseases and drug types (generics/originators). The objectives of this study are to estimate the price elasticity of medicine demand. First, we explore the drug price sensitivity mechanism under the framework of physician agency. Second, we estimate the price elasticity of demand for different medicine types (generic/originator) and different diseases in China.

## Materials and Methods

### Conceptual Framework

Several major institutional features in the Chinese pharmaceutical market differentiate this study’s setting from that in developed countries. First, before 2015, the retail price (*P*_*re*_) of medicines covered by the UEBMI was regulated by the Chinese government by a maximum retail price *P*_*max*_ under the cost-plus approach. The *P*_*re*_ was set on the basis of the ex-factory price (*P*_*ex*_), bidding price (*P*_*bi*_), and markup, and it was limited by the *P*_*max*_ (Zhao and Wu, 2017). To obtain access to public hospitals, pharmaceutical manufacturers participated in the government’s bidding and procurement system to obtain the *P*_*bi*_ (as detailed in the next paragraph). Public hospitals purchased medicines at price *P*_*bi*_. However, they were allowed to make marginal profits (*M*) by selling medicines to their patients under the markup policy framework. The markup policy originated in the 1950s when the healthcare system was welfare. Since then, the government pays the physicians’ salaries and the physicians are not allowed to earn money from diagnosis and treatment. Thus, the physicians only can earn money from incentives (markup rate) which was through prescribing medicines to patients. The markup rate was a “reasonable zone” of profit margin (*θ*), which did not generally exceed 15% for chemical medicines.^3^ The retail price *P*_*re*_ was never allowed to exceed the maximum retail price *P*_*max*_ set by the government:

$$P_{ex}<\left(P_{bi}+M\right)=P_{re}\leq P_{\max}.$$ (1)

Second, to reduce the economic burden on patients, in 2001, the government established the bidding and procurement system by reducing the distribution channel. Although these bidding systems varied among cities, the policies that informed them were similar. A company winning the bid primarily depended on its bidding price (*P*_*bi*_), competition from other companies, product quality, the company’s reputation, and the ability to engage in public relations. First, *P*_*bi*_ was not related to purchase volume in this process. Several provinces’ hospitals have attempted to independently negotiate drug prices with companies; however, this strategy was not permitted by the government, particularly in Tianjin. Second, in the bidding procurement policy, the procurement volume was based on historical consumption.

Quality differences between originators and generics and quality variations among the generics were common in the Chinese pharmaceutical market (Xinhua News Agency, 2018). In order to improve the quality of generics, the evaluation of quality and efficacy was introduced in 2016. The State Council The People’s Republic of China (2016). However, to compete with their originators and other products, the sales representatives of generic companies encourage physicians to prescribe generic drugs. Generic drug representatives often bribe doctors by giving them a commission upon prescriptions. However, it is very difficult to quantify the effect of sales representatives on the demand for prescription drugs due to strong concealment.

The interplay among patients, doctors, and pharmaceutical enterprises is often observed in China. First, in the Chinese healthcare system, physicians prescribe and dispense drugs. Physicians may choose a drug based not only on its efficacy, safety, or cost-effectiveness but also on the extra profit (*π*) they obtain (Xuan et al., 2010; Zeng et al., 2014). There are two types of profits: one is obtained from the markup policy (*M*) and the other is a bribe from the companies’ sales representatives (Eq. 2) which is unobservable. The markup policy could change physicians’ prescribing preference, leading them to prefer expensive drugs, especially for the originators, which can generate higher profits (Jiang and Fan, 2002; Gao et al., 2009; Lu et al., 2011). However, if the bribe utility is larger than that of the markup policy, the physicians may choose the generics. Thus, the double profits that doctors can obtain from prescriptions may be defined as the profit margin related to the markup rate (*θP*_*bi*_) and the illegal rebate (*αP*_*bi*_) (Eq. 2).

We assume a linear relationship between the bidding price and the rebate rate *α*. Medical companies tend to receive a fixed amount of money for the rebate:

$$\Pi =\theta P_{bi}+\alpha P_{bi}=\frac{\theta P_{re}}{\left(1-\theta \right)}+\frac{\alpha P_{re}}{\left(1-\theta \right)}.$$ (2)

Given the institutional features of the Chinese pharmaceutical market, we adopt the dynamic demand function shown in Eq. 3 to model this dynamic process. The product-level demand for prescription drugs (Q_t_) can be expressed as follows:

$$Q_{t}=f\left(Q_{\mathrm{t-1}}\mathrm{,\Pi ,}P_{\mathrm{re}}\mathrm{,D,X}\right).$$ (3)

We hypothesize that the historic consumption volume (*Q*_*t-1*_) of the drug, the profit obtained by physicians (*π*), the retail price (*P*_*re*_), the drug quality and efficiency difference (*D*), and other factors (*X*) will affect the demand for the prescription drug *Q*_*t*_. *X* represents other determinants of the demand for prescription drugs, such as the prices of other competing products (*P*_*c*_) and the UEBMI classification policy. Drugs covered by the UEBMI can be classified as Class A or Class B. Drugs in UEBMI Class A are considered essential and clinically necessary, and their prices are lower than those of Class B drugs.

We assume a linear relationship between *D* and *P*_*re*_. *D* is approximately equal to *γP*_*re*_, and *γ* is positive because price differences are generally used as a proxy for differences in quality.

Based on Eqs 2, 3, the effect of retail price on the demand for drugs can be explained as follows:

$$\frac{\partial Q_{t}}{\partial P_{re}}=\frac{\partial f}{\partial P_{re}}+\frac{\partial f}{\partial \pi }\frac{\partial \pi }{\partial P_{re}}+\frac{\partial f}{\partial D}\frac{\partial D}{\partial P_{re}}=f_{1}+\frac{1}{1-\theta }f_{2}\left(\theta +\alpha \right)+f_{3}\gamma ,$$ (4)

where *f*_*1*_ *= ∂f/∂P*_*re*_, *f*_*2*_ *= ∂f/∂π*, and *f*_*3*_ *= ∂f/∂D*. In Eq. 4, the sign on *f*_*1*_ is negative if the demand law holds. The sign on *f*_*2*_ is positive if physicians’ prescription choices are positively affected by profit margins. The sign on *f*_*3*_ is positive if physicians’ prescription choices are positively affected by the medicine quality. Hence, *θ*, *γ*, and *α* are positively correlated with the absolute value of the drug price elasticity.

The illegal rebate rate *α* plays a significant role in balancing the margin profit difference for physicians between generic and original drugs. Thus, *α*_*g*_ is generally higher than *α*_*o*_ in China. For drug quality, the following relationship can be derived:

$$\gamma =\frac{\text{Δ}D}{\text{Δ}P}\;.$$ (5)

We assume that *γ*_*g*_ and *γ*_*o*_ are the coefficients on the product quality to medicine price ratio for the generic and originator, respectively. Similar to the profit of markup, the product quality is higher if the retail price is higher; hence, *γ* is positive. We assume that the relative change in the product quality to medicine price ratio for the originators is higher than that of the generics. Thus, at the same level of price change for the medicine or the same level of R&D investment, the originator companies could guarantee higher therapeutic value and quality products than generic companies; hence, *γ*_*g*_ < *γ*_*o*_. Thus, we assume that

$$\theta_{g}=\theta_{o},\;\alpha_{g}>\alpha_{o},\;\gamma_{g}<\gamma_{o}.$$ (6)

A crucial implication of Eqs 4, 6 is that the incentive to earn profit reduces the price sensitivity of demand for drugs, and the magnitude of this effect depends on θ and rebate rate *α*. Physicians tend to prescribe medicines that provide them with larger incentives. The mechanism of the rebate rate *α* is similar to the effects of advertising, which “stick” the consumer’s loyalty to the brand. The incentives, the rebate rate, and the quality of the medicine affect the price elasticity of demand. Physicians and patients tend to prefer originators when the originator and the generic drugs have the same price.

We cannot directly test whether the profit margin, bribery rebate, and drug quality affect price sensitivity. However, we can indirectly test these effects by measuring the price elastics for generics and originators. If we do not consider the effect of quality, the price sensitivity of the demand for the generics and originators reads as in Eq. 6. However, the drug quality should be accounted for, and the coefficients *γ*_*g*_ and *γ*_*o*_ should be considered for a price elasticity sensitivity test. If we do not consider the stimulation of economic interests, we only address the impact of quality differences on drug price elasticity. Then, the absolute value of the price in Eqs 7.1, 7.2 reads as

$$\left|\frac{\partial Q^{g}}{\partial P_{re}^{g}}\right|<\left|\frac{\partial Q^{o}}{\partial P_{re}^{o}}\right|\;as\;\left(\theta_{g}+\alpha_{g}\right)>\left(\theta_{o}+\alpha_{o}\right),$$ (7.1)

$$\left|\frac{\partial Q^{g}}{\partial P_{re}^{g}}\right|>\left|\frac{\partial Q^{o}}{\partial P_{re}^{o}}\right|\;as\;\gamma_{g}<\gamma_{o}.$$ (7.2)

From a theoretical perspective, it is difficult to judge the price elasticity between generic and original drugs; its absolute value depends on the relationship between economic benefits and quality differences.

### Data Sources

The primary data were acquired from a randomly drawn 30% sample of the UEBMI database of Tianjin City over the period 2008–2010.^4^ By 2010, 5.1 million members or approximately 39% of registered residents had enrolled (National Bureau of Statistics of the P.R. of China, 2012). The claims data encompass detailed demographics, dates of treatment, treatment received, drug identification number, the unit price of drugs (price per tablet or capsule), the quantity of the drug consumed, and the manufacturer’s name. The Anatomical Therapeutic Chemical (ATC)/Defined Daily Dose (DDD) drug classification system, established by the WHO (World Health Organization, 2017), was used to identify the therapeutic classes and DDDs. For medicines without DDD, we calculated the DDD by ourselves according to the drug instructions. The policy information was retrieved from official Chinese government websites (Ministry of Human Resources and Social Security of the People's Republic of China, 2017).

The drugs investigated in this study include antibiotics, cardiovascular disease (CVD) agents, and anti-tumor agents for addressing both acute and chronic conditions. These are among the most expensive drugs and account for approximately 50% of the total market share of chemical medicines (Cheng, 2012). The observation unit is the product-year-quarter.^5^ The final sample is an unbalanced panel of 1,539 products from 435 manufacturers, covering 252 separate molecules (Table 1).

**TABLE 1:** Sample size and distribution by therapeutic categories.

Variable	Total		Anti-tumor		CVD		Antimicrobial	
	Generic	Originator	Generic	Originator	Generic	Originator	Generic	Originator
**Number of ATC4**	85	80	17	12	33	17	35	17
**Number of ATC5**	206	46	36	18	78	28	92	34
**Products**	1,407	132	96	24	359	49	952	59
**Manufacturers**	368	67	52	21	163	29	223	26

### Variables

The definitions of the empirical variables are shown in Appendix 1. We aggregated the prescription consumption for each visit and admission by quarter, following which we transformed the quantity of drug consumption into DDDs. We used the DDD as a standard unit of measurement and the price per DDD to measure retail prices.

### Estimation Strategy

The empirical strategy for testing the proposed research hypotheses is as follows:

$$LnQ_{i,t}=\beta_{0,i}+\beta_{1,i}LnQ_{i,t-1}+\beta_{2,i}LnP_{re,i,t}+\beta_{3,i}D_{i,t}+\beta_{4,i}X_{i,t}+\varepsilon_{i,t},$$ (8)

where $Q_{i,t}$ denotes the average quantity demanded for the product *i* at time *t* and $Q_{i,t-1}$ represents the drug consumption for the product *i* at time *t−1* (lagged by four quarters). As drug consumption generally shows seasonal variation, we used one year (four quarters) as the lag period. $P_{re,i,t}$ is the retail price for the drug *i* at time *t*. $D_{i,t}$ is the drug quality and efficiency difference. $X_{i,t}$ is a vector of additional explanatory variables that include the price of the competitor (*P*_*c*_), market size (M), and UEBMI policy (*R*), and $\varepsilon_{i,t}$ is an error term. $\beta_{1,i}$ is the dynamic process forming coefficient and is expected to be lower than the one for the stability of the system.

Regarding the proposed dynamic model, two key points are worth highlighting. First, $Q_{i,t-1}$ and market share may be endogenous to the error term. Second, the proposed model does not reflect the price and demand mechanisms observed in Western countries. During the study period, the government regulated the price and discouraged secondary negotiations, and there was no volume discount strategy. Therefore, the retail price was not considered endogenous.

In a dynamic panel context, we used the system generalized method of moments (SYS-GMM) estimator. Given that this estimator is also an instrumental variable approach, we used the Sargan–Hansen test and the Anderson–Rubin tests for over-identifying restrictions and autocorrelation of the residuals, respectively. The first-order and second-order tests were test statistics for first- and second-order autocorrelations of residuals, respectively, under the null hypothesis of no serial correlation. The SYS-GMM regression analysis was conducted using Stata/SE 12.0 (Stata Corp, College Station, TX, United States).

## Results and Discussion

Table 2 presents the statistical results of the sample variables for the three therapeutic drug classes and the total group. The average quarterly sale quantity per drug for originators was always larger than that of a generic drug for the total group and the three therapeutic drug classes (Table 2). CVD drug demand was dominant, followed by anti-tumor and antimicrobial drugs. The price of the original drug is usually about twice that of the generic drug. The highest price belongs to the originator of antimicrobial medicine. The lowest price is for the generic product of CVD.

**TABLE 2:** Statistical description of sample variables from three therapeutic drug classes.

Variable	Total group		Anti-tumor		CVD		Antimicrobial	
	Generic	Originator	Generic	Originator	Generic	Originator	Generic	Originator
	Mean (SD)/N (%)							
**Quantity (Q**)	4,816.40 (35,803.22)	12,537.40 (54,595.19)	976.59 (2,619.50)	1,625.59 (3,759.87)	18,806.55 (72,816.75)	35,397.54 (90,010.64)	599.01 (2014.58)	771.21 (1,362.11)
**Price (P**)	72.17 (134.06)	154.03 (227.10)	102.01 (142.72)	179.12 (223.24)	13.91 (76.40)	31.02 (142.96)	88.34 (142.34)	229.38 (239.59)
$P_{0}^{GM}$	78.37 (167.60)	83.93 (142.97)	114.07 (162.38)	101.17 (123.34)	39.79 (241.46)	36.55 (137.82)	87.47 (132.78)	110.24 (145.03)
$P_{0}^{TM}$	81.34 (101.38)	76.64 (114.74)	129.53 (153.40)	62.72 (66.13)	27.75 (56.08)	13.29 (40.70)	94.12 (99.76)	125.05 (138.06)
**M_size**	0.0637 (0.1715)	0.2232 (0.2779)	0.1673 (0.2603)	0.3461 (0.2753)	0.0963 (0.2239)	0.2618 (0.2788)	0.0425 (0.1300)	0.1528 (0.2575)
**UEBMI Class A**	419 (29.78)	19 (14.39)	48 (50.00)	4 (16.67)	124 (34.54)	10 (20.41)	247 (25.95)	5 (8.47)
**DDD**	1,468.99 (2,337.36)	1,006.12 (2,350.52)	152.34 (298.83)	280.61 (801.02)	107.28 (198.65)	77.61 (81.68)	2,115.25 (2,600.05)	2072.37 (3,180.58)
**Pack size**	12.97 (23.35)	8.14 (12.85)	10.21 (22.39)	8.71 (21.23)	25.72 (31.21)	12.78 (12.63)	8.44 (17.50)	4.07 (5.20)
**Oral normal**	567 (40.30)	60 (45.45)	20 (20.83)	5 (20.83)	232 (64.62)	34 (69.39)	315 (33.09)	21 (35.59)
**Oral sustained**	49 (3.48)	7 (5.30)	0 (0)	0 (0)	37 (10.31)	6 (12.24)	12 (1.26)	1 (1.69)
**Powder for IV**	468 (33.26)	40 (30.30)	39 (40.63)	11 (45.83)	30 (8.36)	3 (6.12)	399 (41.91)	26 (44.07)
**Solution for IV**	323 (22.96)	25 (18.94)	37 (38.54)	8 (33.33)	60 (16.71)	6 (12.24)	226 (23.74)	11 (18.64)

Note: DDD, defined daily dose. UEBMI, Urban Employees’ Basic Medical Insurance.

Table 3 presents the coefficient estimates of the relationship between several variables and demand for the three therapeutic drug classes, while Table 4 presents the coefficient estimates for the generic and originator sub-samples. The Hansen J statistic indicates that the regression does not suffer from over-identification problems. Furthermore, the second-order statistics yielded evidence of the “no serial correlation” assumption.

**TABLE 3:** Estimates of the demand by therapeutic drug classes.

	Dependent variable: log(Q)					
	Specification (1)			Specification (2)		
	Anti-tumor	CVD	Antimicrobial	Anti-tumor	CVD	Antimicrobial
**L1.logQ**	0.356***	0.0896***	0.213***	0.337***	0.0667**	0.175***
	(0.0471)	(0.0275)	(0.0156)	(0.0447)	(0.0272)	(0.0154)
**L2.logQ**	0.108**	−0.00190	0.0355***	0.114**	−0.0186	0.0240***
	(0.0469)	(0.0258)	(0.00875)	(0.0451)	(0.0253)	(0.00828)
**L3.logQ**	0.192***	0.00953	0.0298***	0.190***	0.00727	0.0251***
	(0.0544)	(0.0252)	(0.00841)	(0.0524)	(0.0244)	(0.00780)
**L4.logQ**	0.0104	0.0805***	0.0514***	0.0166	0.0733***	0.0493***
	(0.0526)	(0.0261)	(0.00814)	(0.0505)	(0.0254)	(0.00752)
**Log(P)**	−0.192**	−1.100***	−0.695***	0.541	−0.713***	−2.062***
	(0.0865)	(0.149)	(0.0314)	(0.561)	(0.207)	(0.216)
Log(P)*Generic	—	—	—	−0.716	−0.630***	1.385***
	—	—	—	(0.535)	(0.242)	(0.218)
${\mathrm{Log}}^{\left(P_{c}^{GM}\right)}$	0.0668	0.153	0.141***	0.0590	0.182	0.170***
	(0.0507)	(0.148)	(0.0500)	(0.0491)	(0.144)	(0.0464)
${\mathrm{Log}}^{\left(P_{c}^{TM}\right)}$	0.209***	0.444***	0.277***	0.189***	0.390***	0.239***
	(0.0529)	(0.120)	(0.0221)	(0.0538)	(0.118)	(0.0214)
**Log(M_size)**	0.392***	0.712***	0.712***	0.428***	0.745***	0.729***
	(0.0478)	(0.0388)	(0.0226)	(0.0484)	(0.0378)	(0.0209)
**UEBMI Class A**	−0.375	−4.194***	−2.997***	−0.401	−4.675***	−2.628***
	(0.378)	(0.532)	(0.188)	(0.363)	(0.541)	(0.184)
**Generic**	−0.449	−0.668	5.293***	0.0458	−1.395***	1.954***
	(0.402)	(0.428)	(0.302)	(0.517)	(0.499)	(0.596)
**q1**	−0.0880	−1.350***	0.0155	−0.0778	−1.418***	0.0220
	(0.0886)	(0.105)	(0.0183)	(0.0852)	(0.104)	(0.0169)
**q2**	−0.113	−2.023***	−0.0361*	−0.123	−2.074***	−0.0240
	(0.0843)	(0.0928)	(0.0206)	(0.0994)	(0.0921)	(0.0191)
**q3**	−0.209**	−0.475***	0.0857***	−0.166*	−0.491***	0.0869***
	(0.0967)	(0.0784)	(0.0177)	(0.0961)	(0.0758)	(0.0163)
**Quarter**	Yes	Yes	Yes	Yes	Yes	Yes
**Constant**	2.798***	12.19***	3.230***	0.330	12.09***	10.59***
	(0.605)	(0.765)	(0.382)	(1.970)	(0.741)	(1.206)
**AR2**	0.5917	0.9900	0.1887	0.7307	0.7531	0.0193
**Hansen test**	0.7091	0.1428	0.0564	0.7902	0.3099	0.1677
**Observations**	462	815	3,865	462	815	3,865

Note: UEBMI, Urban Employees’ Basic Medical Insurance. *p < 0.1, **p < 0.05, and ***p < 0.01.

**TABLE 4:** Estimates of the demand by products types (generic vs. originator).

	Dependent variable: log(Q)					
	Anti-tumor		CVD		Antimicrobial	
	Generic	Originator	Generic	Originator	Generic	Originator
**L1.logQ**	0.255***	0.189***	0.0294	0.175***	0.293***	0.141***
	(0.0534)	(0.0620)	(0.0318)	(0.0439)	(0.0209)	(0.0406)
**L2.logQ**	0.0891**	0.266**	−0.0304	0.0745*	0.0420***	−0.0232
	(0.0447)	(0.104)	(0.0283)	(0.0433)	(0.0120)	(0.0423)
**L3.logQ**	0.202***	0.219**	−0.000156	0.0216	0.0385***	0.0628
	(0.0546)	(0.105)	(0.0281)	(0.0547)	(0.0116)	(0.0430)
**L4.logQ**	0.0207	0.00282	0.0557**	0.159**	0.0579***	0.123***
	(0.0549)	(0.0658)	(0.0273)	(0.0671)	(0.0114)	(0.0289)
**Log(P)**	−0.275**	−0.247***	−1.479***	−1.473***	−0.779***	−0.839***
	(0.107)	(0.0907)	(0.165)	(0.188)	(0.0439)	(0.0808)
${\mathrm{Log}}^{\left(P_{c}^{GM}\right)}$	0.0947*	−0.0331	0.356***	−0.398	−0.0146	0.196***
	(0.0572)	(0.0388)	(0.132)	(0.327)	(0.0687)	(0.0593)
${\mathrm{Log}}^{\left(P_{c}^{TM}\right)}$	0.229***	0.800***	0.607***	0.634***	0.288***	0.575***
	(0.0502)	(0.183)	(0.0998)	(0.195)	(0.0323)	(0.0498)
**Log(M_size)**	0.388***	0.654***	0.674***	0.905***	0.580***	0.499***
	(0.0485)	(0.0577)	(0.0400)	(0.0546)	(0.0298)	(0.0331)
**UEBMI Class A**	−0.623	1.313*	−5.722***	−2.483***	−3.063***	−0.330
	(0.385)	(0.722)	(0.679)	(0.534)	(0.261)	(0.546)
**q1**	0.0291	−0.276*	−1.154***	−1.547***	0.0510**	0.199***
	(0.0944)	(0.142)	(0.0996)	(0.270)	(0.0256)	(0.0627)
**q2**	0.0215	−0.0928	−2.022***	−0.591**	0.0407	0.140**
	(0.0891)	(0.149)	(0.0977)	(0.286)	(0.0275)	(0.0606)
**q3**	−0.121	−0.427***	−1.203***	−1.349***	0.117***	0.0554
	(0.0786)	(0.153)	(0.109)	(0.293)	(0.0245)	(0.0595)
**Quarter**	Yes	Yes	Yes	Yes	Yes	Yes
**Constant**	3.140***	0.810	12.32***	11.19***	7.706***	6.248***
	(0.602)	(0.916)	(0.751)	(0.981)	(0.476)	(0.602)
**AR2**	0.5699	0.4929	0.6269	0.2836	0.4822	0.2849
**Hansen test**	0.9865	1.0000	0.6699	1.0000	0.1173	1.0000
**Observations**	363	99	706	109	3,575	290

Note: UEBMI, Urban Employees’ Basic Medical Insurance. ***p < 0.01, **p < 0.05, and *p < 0.1.

In Table 3, the estimated results indicate a significantly negative effect of the price on drug consumption. This result is robust across different specifications. Based on specification (1) in Table 3, the estimated price elasticity is −0.192, −1.100, and −0.695 for anti-tumor, CVD, and antimicrobial drugs, respectively. These price elasticity coefficients indicate that a 10% decrease in the price causes the consumption of the drugs to increase by 1.92, 11.0, and 6.95% in the three groups, respectively. Therefore, we observe that different types of diseases are the main factors leading to considerable differences in drug price elasticity. The absolute value of price elasticity for the anti-tumor drug and antimicrobial drug is less than 1 (inelasticity), while it is more than 1 (rich elasticity) for the CVD drug. For patients who choose treatment, because of the lethality of tumor diseases, the price sensitivity was inelastic. This price inelasticity of demand for cancer patients may also be attributable to limited availability of competing substitute products in the cancer drug markets. Thus, these patients generally have fewer choices. Our findings are consistent with those of Goldman et al. (2004), who reported that the price elasticity of proprietary anti-tumor drugs was very low (−0.1–−0.2).

For antimicrobial drugs, the price elasticity was −0.695 which is between that of the CVD and anti-tumor drugs. Plausible explanations for these results are their emergency use in acute diseases conditions as well as the presence of multiple competing equivalent products in the market. The number of products for antimicrobial drugs is much larger than that for anti-tumor drugs, as shown in Table 1. This may explain why the price elasticity of antimicrobial medicine is much larger than that of anti-tumor drugs. Unlike antimicrobial or anti-tumor drugs, CVD drugs are typically needed for the remainder of the patient’s life, making the patient think about purchasing intermittently, which could also be contributing to the low patient adherence to CVD treatment in China (Xu et al., 2012). In addition, the availability of multiple alternative CVD drugs makes them switch the demand for a specific product type. Furthermore, previous studies confirmed that the price elasticity of medical spending varies with the type of care (Duarte, 2012; Fukushima et al., 2016).

In specification (2) in Table 3, we added an interactive variable Log(P)*Generic into our model. This interactive variable was utilized to explore the final interaction result of quality and unfair competition through the comparison of price elasticity between the generic and the originator. The coefficients of the interactive variable for anti-tumor and CVD drugs were −0.716 and −0.630, respectively. This means that the absolute price elasticity of generic medicine is larger than that of originator medicine for anti-tumor and CVD drugs. Based on our model in Eqs 6, 7, we believe that the low quality of generic medicine contributes to the rise in price elasticity. Even though doctors may accept illegal rebates from the companies, the poor quality makes the doctors prefer originators. Moreover, for the group of antimicrobial drugs studied, the coefficient of the interactive variable is positive and equals 1.385. The value is positive, which will allow the absolute value of price elasticity of generic medicine to be smaller. We believe that this is because the illegal rebate effect dominated. There are too many generic products in the antimicrobial group compared to the other two groups. The competition between the companies forces illegal rebates to take place. Although a quality difference also occurs in this group, the illegal rebates for generic drugs make the drug demand less sensitive to price than in the case of originator drugs. Our result is consistent with that of previous studies that have explored medicine utilization from the reasonable use perspective (Currie et al., 2011; Currie et al., 2014).

Our sub-sample analysis (Table 4) shows that the value of price elasticity is irregular between generic and originator drugs within therapeutic classes, which is consistent with the result of Table 3. For the anti-tumor sub-group, the coefficients were −0.275 vs. −0.247 (generic vs. originator, *p* < 0.001). For CVD drugs, the coefficients were −1.479 vs. −1.473 (generic vs. originator, *p* < 0.001). For the antimicrobial drugs, the coefficients were −0.779 vs. −0.839 (generic vs. originator, *p* < 0.001). The absolute value of price elasticity of demand for anti-tumor and CVD generic medicine was larger than that of the originators, while for the antimicrobial drugs, the value was smaller. These sub-group analysis results are consistent with the results acquired from the interactive variable methods (Tables 3 and 4).

Our results of price elasticity of demand confirmed the existence of drug quality differences and illegal kickbacks. Our findings differ from those of Liu et al. (2012) in Taiwan, where price elasticity of generic drugs was smaller than that of originator drugs (−0.549 vs. −1.721, *p* < 0.01). The reason for this is that the physicians in Taiwan could gain a higher countervailing power from the suppliers of generic products compared to the suppliers of brand-name products. Moreover, the overall quality of generic products is better in Taiwan than in the Chinese mainland (Liu et al., 2012).

Our findings support that the quality of generics has now improved following concerns: to reduce medicine expenditure and to promote a good competitive environment. Owing to the poor quality of generic medicines, physicians tend to prefer the originators, which are more expensive and result in higher medical expenditure. Furthermore, illegal rebates will decrease at a large rate if medicine quality is improved. The origin of illegal rebates is the poor quality of medicines from multiple unfair competitors in the market; thus, generic medicines with consistent quality should be available in the market in China. However, the rebate is a kind of marketing strategy which cannot be avoided completely (Kwon et al., 2015). Since the government has introduced the VBP policy in China, they can decide which kind of medicine can be distributed to the hospital in order to avoid illegal rebates. Based on the two policies above, it is believed that the total expenditure on medicine will finally decrease. However, this policy may have negative impact on the pharmaceutical market. On the one hand, monopoly companies may appear and the government may finally lose ability to negotiate prices with the companies since there will be no more than three competitors in the market. The shortage of large price elasticity medicines could occur due to the sudden lowering of the prices. It is reported that the shortage of the CVD medicines has happened in China (Wu, 2016; Sina Medical News, 2020). On the other hand, there will be concerns with shortages if the price of generics is too low to ensure profitability (Dylst et al., 2013).

Our estimates also address cross price effects (Tables 3 and 4). The positive coefficient of cross price effect indicates the difference of substitution effect in different competitive markets. We find that the average price of competitive products in the same therapeutic field usually has a significant positive impact on demand, which indicates that the substitution effect in the same therapeutic field is more extensive than that in the same molecular field. However, this result is contrary to that in Taiwan (Liu et al., 2012). The difference may be related to the difference in drug quality in mainland China. Therefore, in this study, the drug substitution relationship is mainly manifested in the substitution of the same therapeutic field.

With regard to the policies related to demand, the estimated coefficient of the lagged dependent variables from Q1 to Q4 indicates that drug consumption adjusted to a new equilibrium according to their historical consumption (Tables 3 and 4). The present study further highlights the importance of the bidding and purchase system, which has not been emphasized in earlier works. We also found that the demand for drugs is higher if the products are under UEBMI Class B, but the effect of classification is not significant for anti-tumor drugs (Table 3).

We further found that the quantity of drug consumption has a significant positive association with the market share of larger hospitals. This reflects the market-size effect in the sense that the demand for a specific drug is higher if the buyers of the drug are from larger hospitals.

This study should be interpreted with caution, given the following limitations. First, as we used claims data from Tianjin City only, our findings may not be generalizable to other regions. However, insured patients are the main contributors to prescription drug expenditures, and pricing strategies are very similar across all provinces; thus, our findings have significant policy implications. Second, our measure of drug consumption was aggregated; thus, we could not measure the characteristics of patients and physicians individually. Finally, because of data unavailability, we were unable to assess the relationship between drug price and demand over a longer period or the drugs not covered by health insurance.

Our findings have several policy implications. First, our research supports the generic drug consistency evaluation and VBP policy. Under the same quality conditions, market competition can be more sufficient and transparent. Second, we could directly adopt the generic substitution reform when most of the generics in the Chinese pharmaceutical market are bioequivalent to their originators. In other countries such as Sweden, the government directly adopts the generic substitution reform (Godman et al., 2009; Granlund, 2010). However, patients in Sweden pay the difference in price from an originator to a generic if they still wish an originator which rarely happens in practice with such low prices for generics, e.g., utilization of generic venlafaxine was 99.6% of total venlafaxine (DDD basis) (Godman et al., 2013a; Godman et al., 2013b). Under the VBP policy established in China, the heterogeneity of drug elasticity should be fully considered. In particular for the medicines to cure chronic diseases (e.g., CVD drugs), we should prevent the shortage of drugs due to the rich price elasticity. With the advent of the new policy, shortages of some medicines have been reported in China (Wu, 2016; Sina Medical News, 2020).

Third, under the framework of consistent quality, how to scientifically guide physicians to promote inexpensive multiple-sourced medicines still needs to be further studied in China. A previous study carried out in the United Kingdom showed that the demand-side measures are a well-established method to promote the inexpensive multiple-sourced medicines’ substitutions (Martin et al., 2014). Studies have shown that supply-side measures resulted in generic prices as low as 3% of pre-patent loss prices (Godman et al., 2014). In Abu Dhabi, limited demand-side measures led to increased utilization of patent-protected products following the generic reforms (Abuelkhair et al., 2014). This supports the need for multiple coordinated measures to realize considerable savings from generic availability. In South Korea, in order to increase prescribing of generics vs. originators, the government established a price policy which mainly focuses on setting the maximum reimbursement price (ceiling price) to improve the utilization of generic medicines (Kwon et al., 2015). The results again show that coordinated supply- and demand-side measures are needed to fully realize the savings from inexpensive multiple-sourced medicine availability. Multiple demand-side measures resulted in high international non-proprietary name prescribing. The interests of all stakeholders should be fully considered. Education programs need to be introduced in China to narrow the gap of knowledge and attitude between the physician and patients. Forth, VBP may encourage the physicians seek profit through other ways, and thus, the government needs to establish salary reform for the healthcare system. Under the guidance of macroeconomic policies, a microeconomic incentive mechanism at the personal level of doctors (the most important stakeholders) should also be established to promote the substitution of generic drugs. Finally, measures to increase prescribing of multiple-sourced (generic) drugs over originators and still patented medicines in a class/related class are taken to fully realize savings, especially given an aging population. Though this could lower the burden of the healthcare system, encouraging of the pharmaceutical companies’ willingness to research and develop new medicines should also be considered.

## Conclusion

This study examined the demand for medicines under the regulated market. We assumed that physicians considered the medicine quality as well as the incentive or even illegal rebate as they prescribed medication to patients. Moreover, owing to the bidding and purchasing system, we built a dynamic panel model. Our study has several important findings. First, we found that doctors consider medicine quality differences as well as illegal rebates when they prescribe medicines. Second, we found the heterogeneity of price elasticity of medicine demand. The estimated price elasticities of different medicines (depending on disease type) are different, and the price elasticities of different-type medicines for the same disease are also different. Finally, we demonstrated the dynamic adjustment of drug consumption in China.

## Data Availability

The data analyzed in this study were obtained from the UEBMI database from Tianjin, China. Requests to accessing these datasets should be directed to Jing Wu at jingwu@tju.edu.cn.

## Footnotes

1 The UEBMI system, established in 1998, is one of the three basic medical insurance systems in China. Mandatory participation is planned for all employees in the formal sector. The other two government-run medical insurance programs in China’s universal basic medical insurance system are the Urban Residence Basic Medical Insurance (URBMI) and the New Rural Cooperative Medical Insurance (NRCMI). The reimbursement drug list for the URBMI is almost identical to that of the UEBMI but also includes pediatric medicines. To date, the NRCMI does not feature a national reimbursement drug list, and every province develops its own drug list based on the UEBMI list. The copayment policy is established in the health insurance system in China. The copayment is carried out if the cost of one single medical service is higher than the minimum copayment standard and lower than the highest limited amount of money. The rate of payment for the healthcare system and individuals in the range is as follows: 70% are paid by the healthcare system and 30% by the employed individuals, while 80% are paid by the healthcare system and 20% by the retired individuals.

2 Generic substitution is one of the main measures to control medicine expenditure worldwide. However, there still is a hot debate on the generic substitution in the academic since the bioequivalent does not come through strongly enough to combat the negative comments on generics.

3 The markup policy was canceled in 2015 in China.

4 The project was collaborated with the Tianjin Municipal Medical Insurance Bureau, and the end year was 2010. Thus, our data were from 2008 to 2010. However, it is still meaningful for making drug policies nowadays in China. First, after 2015, the Chinese government gradually established the volume-based purchasing policy for the generic medicines. The medicines obey the price–quantity relationship, and the price could be reduced through purchasing large volume of the medicine. Under the background of the new reform, the price elasticity of medicine study is very important for controlling the medicine expenditure and guarding the medicine supplication. Second, even though some policies have been changed in China nowadays, including removing markup and the generic–originator bioequivalence testing policies, the behavior of the physician’s prescribing pattern could not be changed rapidly. Third, there is still no research about the medicine price elasticity results for bidding purchasing medicine which is a typical purchasing process in China. Thus, the study of medicine’s price–volume relationship is still helpful for the improvement of generic substitution, insurance of the supplication of medicine, and saving medicine expenditure nowadays.

5 The products vary according to the manufacturer, active ingredient, formulation, strength, and pack size.

## Appendix 1

**Key variable names and definitions.**

**Table:**

Variable	Definition	Code
**Quantity (Q)**	Quantity of drug consumption measured in terms of defined daily doses (DDDs)	Continuous variable
**Price (P**)	Retail price per DDD (in CNY)	Continuous variable
$P_{c}^{GM}$	Mean retail price per DDD of other competing products within the same generic market (in CNY)	Continuous variable
$P_{c}^{TM}$	Mean retail price per DDD of other competing products within the same therapeutic market (in CNY)	Continuous variable
**M_size (M)**	Market share of products purchased by secondary and tertiary hospitals	Continuous variable
**UEBMI Class A**	Dummy = 1 for UEBMI Class A drug	1 = yes, 0 = no
**DDD**	Defined daily dose	Continuous variable
**Pack size**	Average number of standard units per pack	Number
**Oral normal**	Oral normal-release form	1 = yes, 0 = no
**Oral sustained**	Oral sustained-release form	1 = yes, 0 = no
**Powder for IV**	Powder form	1 = yes, 0 = no
**Solution for IV**	Intravenous injection	1 = yes, 0 = no
